# Greater Strength Gains after Training with Accentuated Eccentric than Traditional Isoinertial Loads in Already Strength-Trained Men

**DOI:** 10.3389/fphys.2016.00149

**Published:** 2016-04-27

**Authors:** Simon Walker, Anthony J. Blazevich, G. Gregory Haff, James J. Tufano, Robert U. Newton, Keijo Häkkinen

**Affiliations:** ^1^Department of Biology of Physical Activity and Neuromuscular Research Center, University of JyväskyläJyväskylä, Finland; ^2^School of Medical and Health Sciences, Centre for Exercise and Sports Science Research, Edith Cowan UniversityJoondalup, WA, Australia; ^3^Institute of Human Performance, The University of Hong KongHong Kong, China

**Keywords:** eccentric-overload, hypertrophy, voluntary activation, cross-sectional area, resistance training, M-wave

## Abstract

As training experience increases it becomes more challenging to induce further neuromuscular adaptation. Consequently, strength trainers seek alternative training methods in order to further increase strength and muscle mass. One method is to utilize accentuated eccentric loading, which applies a greater external load during the eccentric phase of the lift as compared to the concentric phase. Based upon this practice, the purpose of this study was to determine the effects of 10 weeks of accentuated eccentric loading vs. traditional isoinertial resistance training in strength-trained men. Young (22 ± 3 years, 177 ± 6 cm, 76 ± 10 kg, *n* = 28) strength-trained men (2.6 ± 2.2 years experience) were allocated to concentric-eccentric resistance training in the form of accentuated eccentric load (eccentric load = concentric load + 40%) or traditional resistance training, while the control group continued their normal unsupervised training program. Both intervention groups performed three sets of 6-RM (session 1) and three sets of 10-RM (session 2) bilateral leg press and unilateral knee extension exercises per week. Maximum force production was measured by unilateral isometric (110° knee angle) and isokinetic (concentric and eccentric 30°.s^−1^) knee extension tests, and work capacity was measured by a knee extension repetition-to-failure test. Muscle mass was assessed using panoramic ultrasonography and dual-energy x-ray absorptiometry. Surface electromyogram amplitude normalized to maximum M-wave and the twitch interpolation technique were used to examine maximal muscle activation. After training, maximum isometric torque increased significantly more in the accentuated eccentric load group than control (18 ± 10 vs. 1 ± 5%, *p* < 0.01), which was accompanied by an increase in voluntary activation (3.5 ± 5%, *p* < 0.05). Isokinetic eccentric torque increased significantly after accentuated eccentric load training only (10 ± 9%, *p* < 0.05), whereas concentric torque increased equally in both the accentuated eccentric load (10 ± 9%, *p* < 0.01) and traditional (9 ± 6%, *p* < 0.01) resistance training groups; however, the increase in the accentuated eccentric load group was significantly greater (*p* < 0.05) than control (1 ± 7%). Knee extension repetition-to-failure improved in the accentuated eccentric load group only (28%, *p* < 0.05). Similar increases in muscle mass occurred in both intervention groups. In summary, accentuated eccentric load training led to greater increases in maximum force production, work capacity and muscle activation, but not muscle hypertrophy, in strength-trained individuals.

## Introduction

Both the maintenance and improvement of strength and muscle mass are important goals of physical training interventions in a variety of populations (Voet et al., 2013; Stewart et al., 2014), with resistance training being the most popular method for achieving these outcomes. While it should be acknowledged that distinctly different training programs have led to similar increases in strength and muscle mass during short-term training (Burd et al., 2012; Cadore et al., 2014), training intensity has been observed in some studies to be a key factor mediating adaptive responses (Anderson and Kearney, 1982; Campos et al., 2002; Wernbom et al., 2007). In a review of the literature (Fry, 2004), training intensity could explain ~18–35% of the variance in hypertrophy, and was also an important factor in maximum strength gain.

Training using mechanically driven isokinetic devices allows the individuals to perform (near)maximal contractions during both concentric and eccentric actions. Studies utilizing these devises have shown that performing concentric-only or eccentric-only actions in previously untrained subjects has led to similar gains in maximum isometric and/or concentric force production (Seger and Thorstensson, 2005; Franchi et al., 2015) and that training with fast eccentric-only actions can lead to increased torque during fast concentric actions (Paddon-Jones et al., 2001). Nonetheless, training with eccentric-only actions is thought to be particularly beneficial for muscle hypertrophy compared to concentric-only actions (Higbie et al., 1996; Vikne et al., 2006). Furthermore, studies have shown that the addition of eccentric actions to concentric training has resulted in significant improvements in force production capacity when compared to training with concentric actions alone (Häkkinen and Komi, 1981; Colliander and Tesch, 1990; Dudley et al., 1991). On the other hand, some studies have shown a reduced efficacy for eccentric-only training to elicit improvements in concentric force production (Komi and Buskirk, 1972; Higbie et al., 1996; Hortobágyi et al., 1996; Reeves et al., 2009; Roig et al., 2009), which has led to suggestions of action-specific adaptations due to training with eccentric-only actions.

In contrast to the above mentioned studies, where the subjects can produce a (near)maximal contraction, traditional isoinertial resistance training consists of lifting (concentric), and lowering (eccentric) an identical externally-imposed load. For example, traditional isoinertial resistance training methods, where the same absolute load is lifted and then lowered, may not provide an optimal stimulus in the eccentric phase of the lift. Conceptually, the potential sub-optimal loading during the eccentric phase with traditional isoinertial resistance training is based upon data showing that peak force production capacity is greater during eccentric muscle actions (Katz, 1939). Hence, the relative load used during the eccentric phase in traditional isoinertial resistance training is less than during the concentric phase. This submaximal loading results in lower motor unit recruitment and firing rates during the eccentric phase (Søgaard et al., 1996), which would trigger less sarcoplasmic calcium release and possibly a lesser stimulus for myocellular adaptation (Gehlert et al., 2015). This may be one reason why experienced strength trainers seek alternative training methods in order to further increase strength and muscle mass. Thus, the development of other training strategies to overcome the limitations of traditional resistance training is warranted.

One such strategy may be the use of “accentuated eccentric loading” resistance training, where traditional concentric-eccentric resistance training is performed but an additional external load is imposed during the eccentric phase. Over seven consecutive days, Hortobagyi and colleagues observed greater improvements in both eccentric and isometric, but not concentric, knee extension force production in young (Hortobágyi et al., 2001), and older women (Hortobágyi and DeVita, 2000) following accentuated eccentric load training, which were accompanied by increased vastus lateralis muscle activity (i.e., EMG amplitude). Subsequently, 5 weeks of training using a flywheel system resulted in two-fold increases in quadriceps muscle volume in the accentuated eccentric load group compared to traditional isoinertial resistance training (+6.2 vs. +3.0%), although no statistically significant differences between groups were observed (Norrbrand et al., 2008). More recently, Friedmann-Bette et al. (2010) reported increases in squat jump performance and type IIx fiber cross-sectional area after 6 weeks of accentuated eccentric load in concurrently-training male athletes. In high level volleyball players, using accentuated eccentric loading prior to vertical jumping led to improved countermovement jump performance whereas no change occurred in the group using body weight only (Sheppard et al., 2008). Longer term studies (e.g., 12–16 weeks) in elderly subjects investigating elongated eccentric phase (Dias et al., 2015) and unilateral eccentric following bilateral concentric actions (Raj et al., 2012) showed similar improvements to traditional resistance training in various functional capacity tests. Furthermore, greater increases in strength were observed in some resistance exercises but not others compared to traditional resistance training, and any potential benefit of accentuated eccentric loading did not appear to influence functional capacity test results (Nichols et al., 1995). Nonetheless, no studies have explicitly examined the effects of accentuated eccentric load resistance training aiming to improve maximum strength and muscle mass.

The above studies indicate a potential for accentuated eccentric loading to improve strength and muscle mass, particularly during short-term interventions in experienced strength trainers, but they do not allow strong conclusions to be drawn about the efficacy of training with accentuated eccentric loads. An additional issue is that little information detailing the neuromuscular adaptations associated with such training has been presented, hence it is unclear which neuromuscular adaptations might be enhanced in comparison to those elicited by traditional forms of resistance training. It is currently unclear, therefore, how this training strategy might augment force production in strength-trained individuals. Consequently, the aim of the present study was to examine the effects of 10 weeks (i.e., 2 × 5-weeks mesocycles) of lower-limb accentuated eccentric load resistance training on maximum force production, muscle activation, and muscle hypertrophy in subjects accustomed to resistance training. We hypothesized that greater increases in muscle strength, activation and size would result from accentuated eccentric loading than traditional isoinertial training in strength-trained men.

## Materials and methods

### Study design

This study utilized a twin-control group design. A standard control group (CON) continued their normal training without supervision but completed the same testing as the accentuated eccentric load-training group (AEL). The subjects in this group all reported using a split-routine training program without specific focus (i.e., all body parts targeted equally) and their aim was to improve overall strength and muscle mass. A second group performed a traditional concentric-eccentric isoinertial training program (TRAD) under the same supervision and dietary conditions as the AEL group. This type of training group may be considered a true control because subjects are exposed to the same study conditions as the experimental group (Newton et al., 1999). The AEL group performed the same training as TRAD but used greater loading during the eccentric phase, as described in detail below. Subjects in TRAD and AEL were instructed to continue with their normal exercise program for the upper limbs while resistance training of the legs was restricted to the present study's training program during the 10-week period.

Before entering the intervention phase of the study, subjects underwent background screening and then attended four laboratory-based sessions, each separated by 3–4 days. During the first visit, the subjects were familiarized with the test procedures and practiced the strength tests. During the following visits, the subjects; (1) completed body composition assessment using dual-energy X-ray absorptiometry (DXA) after an overnight fast, (2) had their quadriceps cross-sectional area (CSA) measured using ultrasound imaging, and (3) performed unilateral isometric and isokinetic knee extension tests.

**Figure 1:**
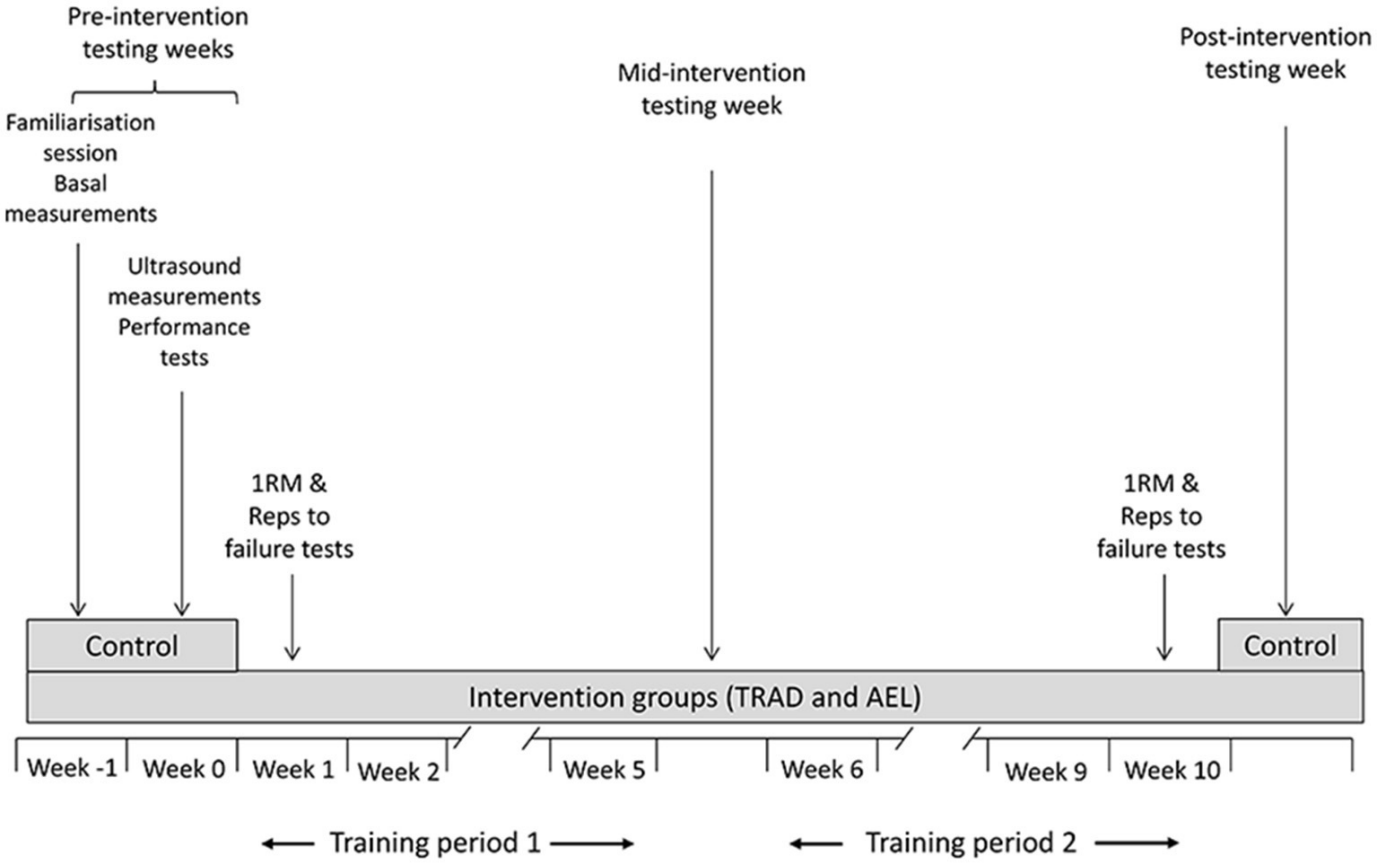
**Overall study design including all measurement points**.

These three test sessions were repeated by the intervention groups (TRAD and AEL) after 5 and 10 weeks of training and by the standard control group (CON) after 10 weeks (Figure 1). After 5 and 10 weeks of training, session 1 (DXA) was completed 3–4 days after the last training session with sessions 2 (ultrasound) and 3 (strength tests) being completed in 1–3 days intervals so that the strength tests always took place 7 days after the last training session.

### Subjects

Thirty-three healthy, young men agreed to participate in the study after being informed of all the risks and providing signed informed consent. The subjects had previous experience of resistance training (2.6 ± 2.2 years, range: 0.5–6 years). Five subjects (2 TRAD and 3 AEL) withdrew from the study during the 10-week training period (1 exercise-induced headache, 2 muscle soreness, 1 other commitments, 1 gave no reason). Therefore, 28 subjects (age 21 ± 3 years, height 177 ± 7 cm, body mass 75 ± 11 kg) completed all study requirements. The study methods were approved by the Human Research Ethics committee at Edith Cowan University and conducted according to the Declaration of Helsinki.

The subjects were matched (into groups of three subjects) pre-intervention by body mass and neuromuscular strength, and then randomly assigned to AEL (age 21 ± 2 years, height 179 ± 8 cm, body mass 76 ± 11 kg; *n* = 10), TRAD (age 21 ± 2 years, height 178 ± 7 cm, body mass 78 ± 12 kg; *n* = 10), or CON (age 24 ± 4 years, height 176 ± 3 cm, body mass 75 ± 7 kg; *n* = 8). Subjects completed training diaries throughout the study to document all exercise performed outside of the study.

### Training intervention

**Figure 2:**
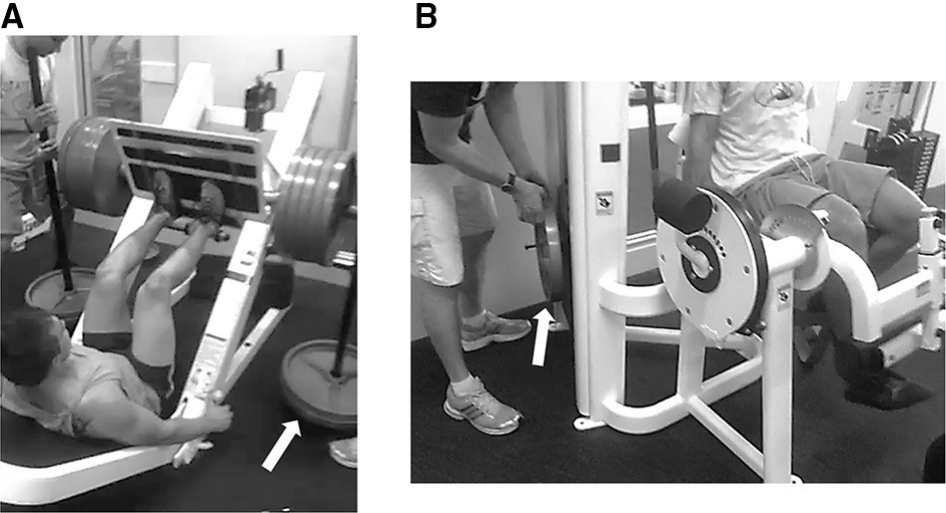
**Training equipment to allow accentuated eccentric loading**. Inclined leg press with weight-releasers **(A)** and manually loaded/unloaded weight plates on the knee extension device **(B)**.

TRAD and AEL engaged in two 5-week training periods where training was performed twice a week (Monday and Thursday or Tuesday and Friday, to allow at least 48 h recovery between training sessions). Training consisted of three sets of 6-RM (session 1) and 10-RM (session 2) bilateral leg press and unilateral knee extension and flexion exercises. Since the aim of training was to increase maximum strength and muscle size, the repetition ranges chosen for each session utilized the stimulating effect of high force and high volume training, respectively. Each subject had their training and testing time standardized throughout the study (±1 h). TRAD performed the exercises with the same load for both concentric and eccentric phases, while AEL performed the exercises with 40% greater load during the eccentric phase compared to the concentric phase (i.e., eccentric load = concentric load + 40%), which was a similar loading protocol to those used by Brandenburg and Docherty (2002) and Ojasto and Häkkinen (2009). In order for each training session to include a true RM, both TRAD and AEL used loads that elicited concentric failure in at least 1 out of 3 sets with the investigator assisting the subject to complete the set. Custom weight-releasers were used to add the additional eccentric load to the leg press exercise (Figure 2A) while weight plates were manually added and removed by the training supervisor(s) with the use of a custom-built pin for the knee extension exercise (Figure 2B). Both groups performed the concentric and eccentric phases of the lift with a 2:2 s tempo (i.e., 4 s in total), which was monitored by the investigator. Immediately after each training session TRAD and AEL subjects were given a standardized recovery drink containing 23 g of whey protein (8.47 g leucine and 5.08 g isoleucine per 100 g), 3 g of carbohydrate, and 1.6 g of fat (Total+, Vital Strength, PowerFoods International Pty. Ltd., Marrickville, New South Wales, Australia) to maximize the initial protein synthesis response to training and standardize post-exercise nutrition between groups.

### Familiarization session

All subjects completed a familiarization session 1 week prior to performance testing (Figure 1). Strength tests (isokinetic followed by isometric) were performed with the right leg until peak torque values in three consecutive attempts were within 5%. Subjects were positioned in an isokinetic dynamometer (Biodex System 3, Biodex Medical Systems, Shirley, USA) so that their knee joint was in-line with the axis of rotation and inelastic straps were placed across the shoulders, hips, thigh, and ankle to minimize extraneous movement. The limits of motion were set so that each subject performed concentric followed by eccentric knee extensions from a 90° knee angle to a 150° knee angle (straight leg and full knee extension = 180°). Three concentric-eccentric warm-up repetitions were performed at an estimated 50% and then 75% of perceived maximum exertion at 30°.s^−1^ before performing two sets of three maximal repetitions separated by 1 min. Thereafter, the subjects were positioned into a custom-built isometric dynamometer (Edith Cowan University, Joondalup, Australia). The knee angle was set to 110° with a hip angle of 100° and subjects were secured firmly by inelastic straps across the shoulders, hips, and ankle. Each subject was allowed 3–5 practice trials following the commands “push as fast and as hard as you can.” Real-time visual feedback was provided to instruct the subject to rapidly achieve maximum torque and maintain the contraction for 3–4 s.

### Surface electromyography

Bipolar surface electromyogram (EMG) electrodes (2 cm center-to-center inter-electrode distance, MediTrace 200, Kendall, Mansfield USA) were placed on the vastus lateralis (VL), vastus medialis (VM), rectus femoris (RF), and biceps femoris (BF) muscle bellies according to SENIAM guidelines after shaving and lightly abrading the skin. Raw EMG signals were pre-amplified (ZeroWire, Aurion Ltd., Milan, Italy), passed via WiFi to a receiving box, relayed to a 16-bit A/D converter (PowerLab system, AD Instruments Ltd., Bella Vista, Australia) and sampled at an analog-to-digital conversion rate of 2000 Hz at a band width of 10–1000 Hz (common-mode rejection ratio = 100 dB). EMG signals were further filtered using a band width of 10–350 Hz offline then assessed by root mean square (_RMS_) analysis over the 60° range of motion during isokinetic trials. For isometric trials, average EMG_RMS_ amplitude was measured over 500 ms in the torque plateau (including the point of instantaneous peak torque). During analysis, EMG_RMS_ data were averaged for the VL and VM (i.e., VL + VM/2) and superficial quadriceps muscles (i.e., VL + VM + RF/3) for both isokinetic and isometric actions.

### Electrical stimulation procedures (M-wave measurement)

To obtain the maximal M-wave amplitude for EMG normalization (i.e., calculation of EMG:M-wave ratio), single square-pulse (400 V, 200 μs duration) electrical stimulations were given by a constant current stimulator (Model DS7AH, Digitimer Ltd., Welwyn Garden City, UK) to the femoral nerve through 5 cm^2^ self-adhesive electrodes (Hollywog LLC, Chattanooga, USA) placed in the femoral triangle either side of the nerve (identified by palpating and locating the femoral artery). The electrodes were moved slightly until the greatest knee extension twitch torque was achieved with a low stimulation intensity. The intensity was then increased until there were no further increases in peak-to-peak M-wave amplitude of VL, VM, or RF or the torque response (typically 300–500 mA). To ensure maximal activation, an additional 20% current was used to induce three maximum single-pulse twitches. The highest value was used to normalize voluntary EMG data during the knee extension tests.

### Maximum isokinetic knee extension tests

Following a 5-min warm-up using a cycle ergometer at a cadence of 70 rpm with 1 kg of resistance (Monark 818E, Monark Ergomedic, Sweden), the subjects were positioned in the isokinetic dynamometer and firmly secured as described above. One warm-up set of three concentric-eccentric repetitions was performed at 80% of perceived maximum exertion. Thereafter, two sets of three repetitions were performed with maximum voluntary exertion at 30°s^−1^. The velocity of the isokinetic actions was chosen to closely resemble the tempo performed during training. Torque and displacement data were synchronously recorded at a rate of 2000 Hz using LabChart software (version 6.1.3, AD Instruments, Dunedin, New Zealand) for offline analysis. During analysis, torque, and displacement signals were low-pass filtered (20 Hz cut-off frequency; Butterworth 4th order). The concentric and eccentric phases were identified from the displacement data and the highest torque values for each action were obtained for further analysis. Test-retest reliabilities [intra-class correlation coefficient (ICC) and coefficient of variation (CV%)] for concentric and eccentric peak torques were 0.903 and 4.7% and 0.947 and 5.6%, respectively.

### Maximum isometric knee extension tests

Subjects were positioned into a custom-built isometric dynamometer and performed unilateral isometric knee extension trials as described above. Three trials were performed, with a fourth trial being required if the third trial yielded more than 5% greater torque compared to the previous trials. Loud, verbal encouragement was given throughout each trial and real-time feedback was provided to the subjects. Torque data were sampled and filtered as described for the isokinetic trials. Analysis was performed offline and assessed for maximum torque. Test-retest reliability for isometric torque was 0.949 (ICC) and 4.1% (CV%).

Maximum isometric actions with superimposed femoral nerve stimulation were subsequently performed with the torque increasing to a maximum over ~2 s and then being maintained for ~4 s. To determine the stimulation intensity, double-pulse (10 ms inter-pulse interval) stimulations were delivered until a plateau in the torque response was observed (typically 400–700 mA) and then an additional 20% current was given to ensure maximal activation. Three trials were performed with two double-pulse stimulations given at the torque plateau and a double-pulse stimulation given two seconds after relaxation. Only trials that achieved an MVC >95% of the non-stimulation trials were included in further analyses. Prior to data filtering, the torque deflection due to electrical stimulation during contraction and at rest following the contraction was determined. Voluntary activation level (VA%) was estimated by the following formula;

$$\text{VA}\%=[1-\frac{\begin{array}{l} (\text{superimposed twitch torque}\\ \;-\text{maximum voluntary torque}) \end{array}}{\left(\text{resting twitch torque}\right)}]\times 100$$

Test-retest reliabilities (ICC and CV%) for voluntary activation level were 0.69 and 4.4%.

### One-repetition maximum knee extension tests

Intervention group subjects performed a unilateral knee extension one-repetition maximum (1-RM) test with the right leg during the first and last week of training (VR3 leg extension, Cybex International Inc., Medway, USA). Each subject performed 5 min cycling (1 kg load at 70 rpm) as warm-up followed by a series of submaximal warm-up sets (6 reps at an estimated 10-RM load, 3 reps at an estimated 6-RM load, 1 rep at an estimated 3-RM load). Thereafter, single repetitions were performed until the subject could no longer lift the load from the beginning knee angle of ~85° to the required knee angle (~170° knee angle), which was set by a rubber stopper fitted to the device. The last successfully lifted load was recorded as the subject's 1-RM. Test-retest reliabilities (ICC and CV%) for 1-RM were 0.99 and 2.4%.

### Repetition-to-failure tests

Three days after the unilateral 1-RM test, the subjects in the intervention group performed a unilateral knee extension repetition-to-failure test with 75% of their 1-RM load using the right leg. Thus, the same relative load, but different absolute load, was lifted before vs. after the training period. Subjects were instructed to maintain a cadence of 2 s concentric and 2 s eccentric phases, which was monitored by the investigator, and to perform as many repetitions as possible. The test was terminated when the subject was unable to lift the load to the rubber stopper for two successive repetitions and only successful repetitions were used in further analysis. Test-retest reliabilities (ICC and CV%) for 1-RM were 0.872 and 8.7%.

### Muscle cross-sectional area

VL, VM, RF, and vastus intermedius (VI) cross-sectional area (CSA) were assessed using B-mode axial-plane ultrasound (model SSD-α10, Aloka Co Ltd., Tokyo, Japan) using a 10 MHz linear-array probe (60 mm width) in extended-field-of-view mode (23 Hz sampling frequency). The validity and reliability of this method, as well as the general scanning technique have been reported previously (Ahtiainen et al., 2010; Noorkoiv et al., 2010). In the present study, three axial-plane panoramic CSA images were taken each at 33, 50, and 67% femur length measured from the lateral aspect of the distal diaphysis to the greater trochanter. CSA was determined by manually tracing along the border of the each muscle using Image-J software (version 1.37, National Institute of Health, USA). As there is no observable aponeurosis between VL and VI at 67% femur length, these muscles were analyzed by tracing the borders of both muscles combined. The mean of the two closest values was taken as the CSA result for each muscle at each site and used in further analysis. Test-retest reliabilities (ICC and CV%) were 0.94 and 4.2% for VL CSA at 50% femur length, 0.93 and 5.1% for VL + VI CSA at 67% femur length, and 0.77 and 10.2% for VM at 33% femur length.

### Dual-energy x-ray absorptiometry (DXA) body composition measurements

Whole-body and lower limb composition of the intervention groups were assessed using DXA (Hologic Discovery A, Waltham, WA) using previously established, standardized and reliable body positioning procedures (Peiffer et al., 2010). The subjects were positioned supine and the DXA operator manually assisted subjects to straighten their head, torso and pelvis, internally rotate and fixate their legs and feet at 45°. This has been shown to produce a scan/re-scan coefficient of variation below 1% in our laboratory (Peiffer et al., 2010). Inbuilt analysis software (Version 12.4; QDR for Windows, Hologic, Waltham, WA) was used to separate the body into axial and appendicular sections to determine whole body composition and segmental composition of the legs.

### Statistical analyses

Traditional methods were used to calculate means, standard deviations and standard error estimations (IBM SPSS version 20). After tests of normality, significant main effects were assessed by analysis of variance (ANOVA) with repeated measures (3 group × 2 times) with Bonferroni adjustments used as *post-hoc* tests. The exception being EMG data where the gross amplitude cannot be compared between individuals/groups- for this variable, paired *T*-tests determined changes over time. One-way ANOVA was used to determine between-group differences for relative changes over the study period. The alpha level was set at 0.05 and effect sizes (*g*) were calculated according to Hedges' methods (Hedges, 1982), where small (< 0.3), medium (0.3–0.8), and large (>0.8) effect sizes are identified.

## Results

### Maximum concentric and eccentric torque

**Figure 3:**
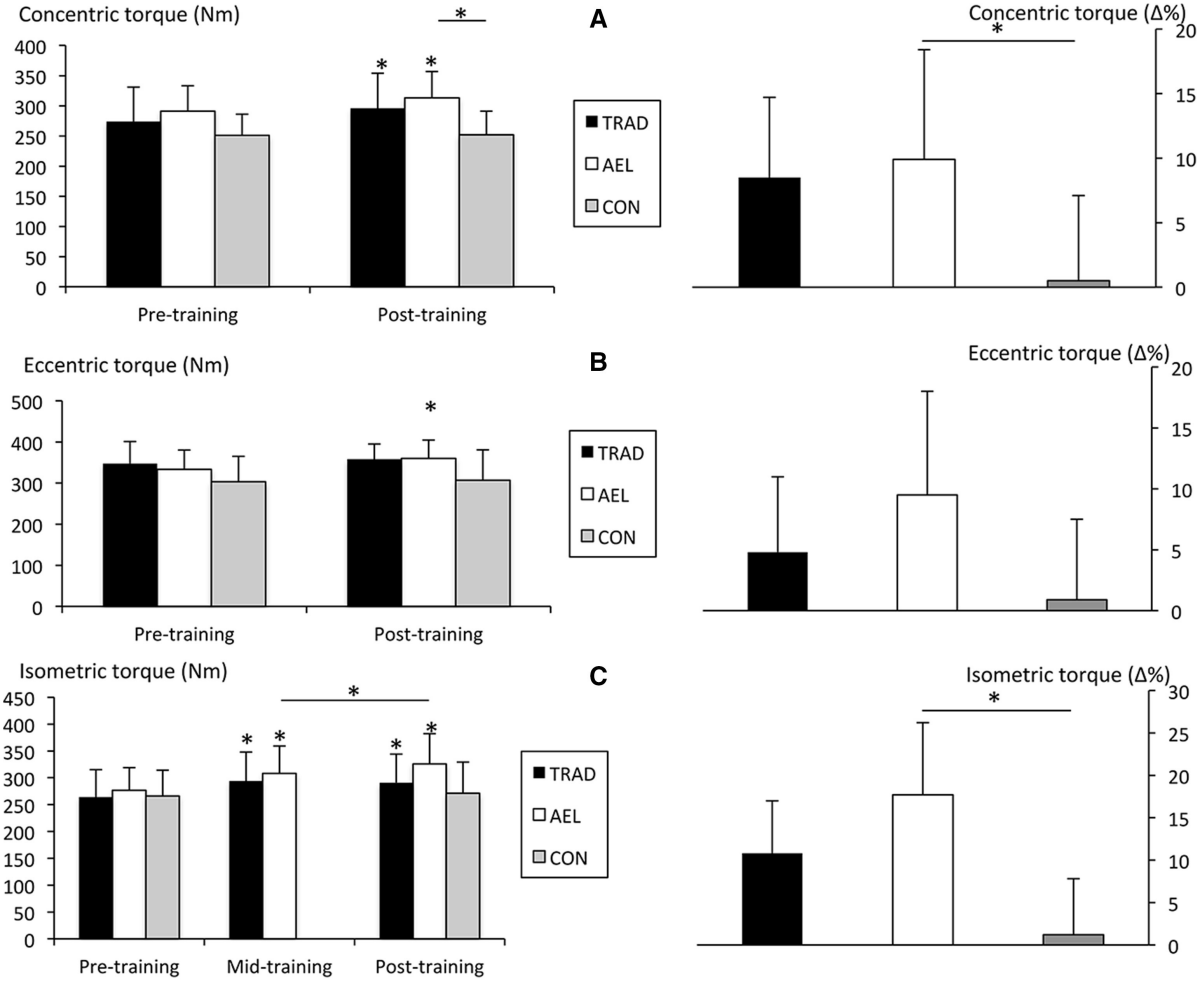
**Absolute and relative changes (mean ± ***SD***) in maximum unilateral isokinetic concentric (A—top panel), eccentric (B—middle panel), and isometric (C—bottom panel) torque production**. ^*^p < 0.05.

There was a significant main effect for time (*F* = 13.4, *p* = 0.001, *g* = 1.64) and a trend toward a group × time interaction (*F* = 2.5, *p* = 0.099, *g* = 0.18) in peak unilateral concentric torque. *Post-hoc* analyses revealed that peak concentric torque significantly increased in both TRAD (PRE: 274 ± 57 Nm, POST: 296 ± 58 Nm, +9 ± 6%, *p* < 0.001, *g* = 0.37) and AEL (PRE: 286 ± 41 Nm, POST: 313 ± 46 Nm, +10 ± 9%, *p* = 0.006, *g* = 0.61) after 10 weeks of training, with no changes in CON (PRE: 251 ± 35 Nm, POST: 252 ± 39 Nm, +1 ± 7%, *p* = 0.81). The relative changes over the study were significantly greater in AEL (*p* = 0.038, 95% confidence interval = 0.4–18.3%) and close to significance in TRAD (*p* = 0.082, 95% confidence interval = −0.8–16.7%) than CON (Figure 3A).

A significant main effect for time (*F* = 7.9, *p* = 0.01, *g* = 1.41), but not group × time interaction (*F* = 1.6, *p* = 0.22, *g* = 0.11), was observed for peak unilateral eccentric torque. Only AEL significantly increased peak eccentric torque after training (PRE: 331 ± 50 Nm, POST: 361 ± 49Nm, +10 ± 9%, *p* = 0.016, *g* = 0.62). However, the relative increase over the study period was not significantly different greater than CON (+1 ± 8%, *p* = 0.17, 95% confidence interval = −2.5–19.7, Figure 3B).

### Maximum isometric torque

Significant main effects for time (*F* = 25, *p* < 0.001, *g* = 2.88) and group × time interaction effect was observed (*F* = 7.63, *p* = 0.003, g = 0.32) in maximum unilateral isometric torque. *Post-hoc* analyses revealed improvements over the 10-week intervention period that were statistically significant in TRAD (PRE: 264 ± 51 Nm, POST: 291 ± 53 Nm, +11 ± 11%, *p* = 0.007, *g* = 0.49) and AEL (PRE: 277 ± 42 Nm, POST: 326 ± 56 Nm, +18 ± 10%, *p* < 0.001, g = 0.95), whereas there was no change in CON (PRE: 266 ± 48 Nm, POST: 271 ± 58 Nm, +1 ± 5%, *p* = 0.34). Also, the AEL group demonstrated significant improvements from week 5 to week 10 (MID: 308 ± 51 Nm, POST: 326 ± 56 Nm, +7 ± 7%, *p* = 0.03, *g* = 0.33), while TRAD did not (MID: 294 ± 54 Nm, POST: 291 ± 53 Nm, –0.8 ± 8%, *g* = −0.06). Relative improvements in isometric torque were significantly greater in AEL than CON over the study period (*p* = 0.001, 95% confidence interval = 5.2–27.8%, Figure 3C).

### One-repetition maximum and repetition-to-failure

For 1-RM strength, there was a significant main effect for time (*F* = 129, *p* < 0.001, *g* = 1.53), but not group × time interaction (*F* = 0.8, *p* = 0.39, *g* = 0.06), over 10 weeks of training in TRAD (PRE: 67 ± 14 kg, POST: 91 ± 18 kg, +36 ± 13%, *p* < 0.001, *g* = 1.38) and AEL (PRE: 71 ± 11 kg, POST: 92 ± 13 kg, +31 ± 13%, *p* < 0.001, *g* = 1.68), with no differences between groups. In the repetition-to-failure test, there was a significant main effect for time (*F* = 11.8, *p* = 0.003, *g* = 0.86) but not group × time interaction (*F* = 1.2, *p* = 0.29, *g* = 0.09), with *post-hoc* tests revealing that the AEL group improved significantly (PRE: 572 ± 166 kg, POST: 716 ± 205 kg, +28 ± 30%, *p* = 0.022, *g* = 0.73) with no change in TRAD (PRE: 526 ± 135 kg, POST: 629 ± 143 kg, +24 ± 34%, *p* = 0.076, *g* = 0.71).

### Quadriceps cross-sectional area

**Figure 4:**
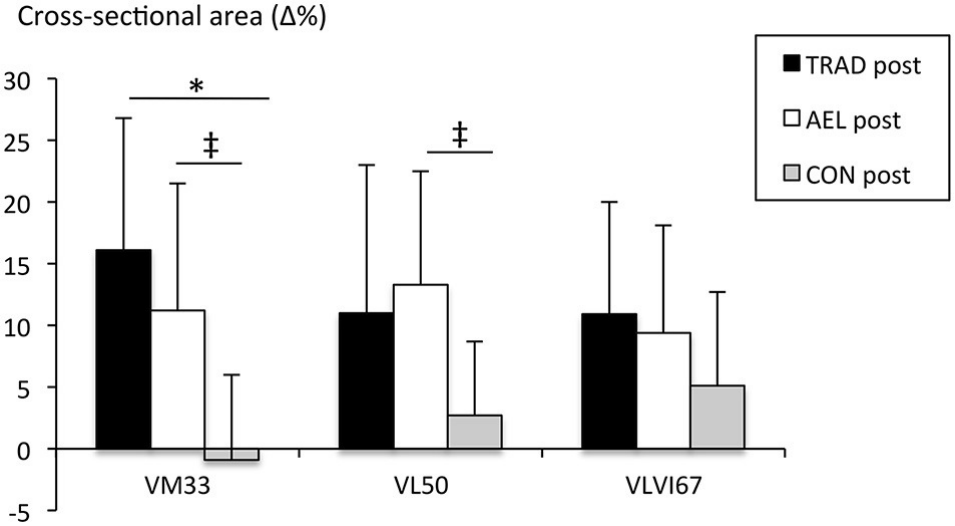
**Relative changes (mean ± ***SD***) in vastus medialis, vastus lateralis and vastus lateralis+intermedius cross-sectional area at 33, 50 and 67% femur length, respectively**. ^*^p < 0.05 between intervention and control groups. ^‡^*p* < 0.1 between intervention and control groups.

A significant main effect for time (*F* = 27.76, *p* < 0.001, *g* = 0.34) and a trend for group × time interaction (*F* = 2.94, *p* = 0.071, *g* = 0.27) were observed for VL at 50% femur length. Increases over the 10-week training period were significant in TRAD (PRE: 23.6 ± 4.6 cm^2^, POST: 25.9 ± 4.5 cm^2^, +11 ± 12%, *p* = 0.019, *g* = 0.48) and AEL (PRE: 24.9 ± 7.8 cm^2^, POST: 27.7 ± 6.7 cm^2^, +13 ± 9%, *p* < 0.001, *g* = 0.36) but not in CON (PRE: 20.6 ± 3.5 cm^2^, POST: 21.2 ± 4.2 cm^2^, +3 ± 6%, *p* = 0.22). One-way ANOVA revealed that relative changes over the study period in VL CSA were close to significance between AEL and CON (*p* = 0.079, 95% confidence interval = −0.9-21.9%, Figure 4).

A significant main effect for time (*F* = 18.8, *p* < 0.001, *g* = 0.51), but not group × time interaction (*F* = 1.7, *p* = 0.20, *g* = 0.11), was observed for VM at 33% femur length. Both TRAD (PRE: 24.0 ± 4.4 cm^2^, POST: 27.7 ± 4.4 cm^2^, +16 ± 11%, *p* = 0.002, *g* = 0.48) and AEL (PRE: 26.6 ± 5.9 cm^2^, POST: 29.1 ± 4.2 cm^2^, +11 ± 10%, *p* = 0.013, *g* = 0.36) significantly increased VM CSA during training. No changes occurred in CON (PRE: 23.8 ± 4.7 cm^2^, POST: 23.6 ± 5.6 cm^2^, *p* = 0.83, −0.9 ± 7%), and the relative changes over time were statistically different compared to TRAD (*p* = 0.005, 95% confidence interval = 4.7–29.4%) and close to significance to AEL (*p* = 0.055, 95% confidence interval = −0.2-24.5%). No changes occurred in CSA at 67% femur length within or between groups.

### Body composition

There were no changes in total-body fat percentage, fat mass or lean mass. However, there was a significant main effect for time (*F* = 12.1, *p* = 0.003, *g* = 0.24), but not group × time interaction (*F* = 2.2, *p* = 0.15, *g* = 0.19), in lean leg mass, where increases in both training groups over 5 weeks (TRAD: +2.6 ± 2.4%, *p* < 0.05; AEL: +3.3 ± 3.9%, *p* < 0.05) and 10 weeks (TRAD: +1.6 ± 2%, *p* < 0.05, *g* = 0.13; AEL: +4.2 ± 4.5%, *p* < 0.05, *g* = 0.31) of training with no between-group differences.

### Isokinetic and isometric muscle activation

Paired *T*-tests revealed that absolute eccentric EMG_RMS_ was significantly increased in AEL over 10 weeks of training for VL and VM (+30 ± 34%, *p* = 0.017, *g* = 0.83) and the superficial quadriceps (+24 ± 34%, *p* = 0.02, *g* = 0.66, Table 1). Similarly, only AEL demonstrated increased absolute EMG_RMS_ after 10 weeks of training for the VL and VM muscles (+49 ± 37%, *p* = 0.007, *g* = 0.99) and the superficial quadriceps (+25 ± 24%, *p* = 0.041, *g* = 0.64, Table 1) during maximum isometric knee extension. Maximum M-wave peak-to-peak amplitude increased significantly in the VL (week 0: 3.1 ± 1.5 mV; week 10: 4.8 ± 1.7 mV, *p* = 0.006) and VM (week 0: 5.0 ± 1.5 mV; week 10: 6.3 ± 0.4 mV, *p* = 0.038) in AEL only. Consequently, once the EMG_RMS_ data was normalized to maximum M-wave, no significant main effects or changes were observed in AEL (Table 1).

**Table 1:** **Mean (± ***SD***) absolute and normalized quadriceps muscle activity (EMG VL + VM + RF/3) during maximum eccentric, isometric, and concentric actions**.

	**Eccentric**				**Isometric**				**Concentric**			
	**Pre-training**		**Post-training**		**Pre-training**		**Post-training**		**Pre-training**		**Post-training**	
	**EMG (mV)**	**%M_max_**	**EMG (mV)**	**%M_max_**	**EMG (mV)**	**%M_max_**	**EMG (mV)**	**%M_max_**	**EMG (mV)**	**%M_max_**	**EMG (mV)**	**%M_max_**
TRAD	0.4 ± 0.2	12 ± 4	0.45 ± 0.1	12 ± 5	0.29 ± 0.1	8 ± 2	0.35 ± 0.1	8 ± 2	0.41 ± 0.1	12 ± 4	0.47 ± 0.1	12 ± 3
AEL	0.42 ± 0.2	12 ± 4	0.54 ± 0.2^*^	13 ± 5	0.34 ± 0.1	10 ± 3	0.42 ± 0.1^*^	10 ± 2	0.42 ± 0.1	13 ± 4	0.57 ± 0.2^‡^	13 ± 5
CON	0.31 ± 0.1	10 ± 5	0.45 ± 0.2	11 ± 3	0.24 ± 0.1	8 ± 2	0.37 ± 0.1	9 ± 2	0.31 ± 0.1	9 ± 2	0.46 ± 0.2^‡^	10 ± 3

* *p < 0.05 vs. pre-training*.

‡ *p < 0.1 vs. pre-training. TRAD, traditional resistance training; AEL, accentuated eccentric load resistance training; CON, control group*.

**Figure 5:**
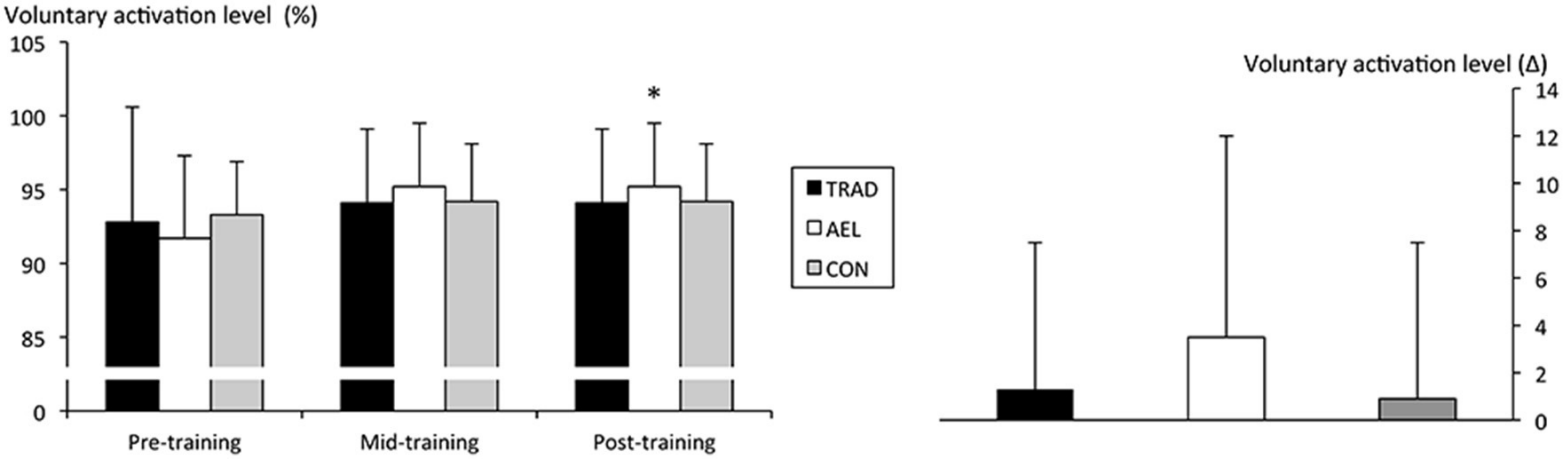
**Voluntary activation level (mean ± ***SD***) during unilateral isometric knee extension**. ^*^p < 0.05 vs. pre-training.

A significant main effect for time (*F* = 23.7, *p* < 0.001, *g* = 0.5), but not group × time interaction (*F* = 0.8, *p* = 0.48, *g* = 0.01), was observed in voluntary activation level measured during isometric action. Increases occurred over 10 weeks of training in AEL only, which was largely observed from week 5 to week 10 (PRE: 91.7 ± 6%, MID: 92.1 ± 8%, POST: 95.2 ± 4%, +3.5 ± 5%, *p* = 0.039, *g* = 0.67). However, between-group differences were not statistically significant (Figure 5).

## Discussion

The aim of this study was to determine the effects of accentuated eccentric load resistance training on lower-limb maximum and repeated force production, muscle activation, and muscle hypertrophy in already strength-trained men. Systematic heavy resistance training led to distinct improvements in neuromuscular performance and knee extensor muscle mass in both TRAD and AEL intervention groups when compared to a continuing, unsupervised (training) control group (CON) within the first 5-week mesocycle. Subsequently, however, accentuated eccentric loading tended to provide a greater training stimulus for strength (isokinetic and isometric), fatigue resistance (repetition-to-failure test) and muscle activation particularly in the second 5-week mesocycle when TRAD elicited no further improvements. These changes resulted in more notable strength and neuromuscular adaptations in AEL compared to CON over the 10-week program.

Maximum force production increased significantly in both AEL and TRAD, with increases of ~30% in 1-RM and ~10% in concentric and ~8% in eccentric strength, as well as ~11% (TRAD) and ~18% (AEL) in isometric torque. These increases in force production are slightly less than those observed in previously untrained individuals (Narici et al., 1989; Häkkinen et al., 1998) but they are larger than improvements reported in other studies in long-term strength athletes (e.g., Ahtiainen et al., 2003). Strength and muscle mass gains are difficult to achieve in experienced strength trainers, even when programming strategies are changed frequently (Häkkinen et al., 1987). The successful effect of training in the present subjects might have been elicited by factors such as the; (1) high intensity loading used (e.g., 6-RM), (2) in-session supervision (including spotting of lifts, encouraged increments in load and assistance provided at concentric failure) and motivation (Mazzetti et al., 2000), and (3) immediate post-training protein consumption, all of which were not provided to the control group. Through the inclusion of CON we were able to establish between-day test reliability and assess potential changes in a representative cohort with continued (i.e., unchanged) training, while the inclusion of traditional isoinertial loading allowed quantification of changes related to training supervision (i.e., the Hawthorne effect), modification of the subjects' current training intensities and volumes, and post-exercise nutrition. Subsequently, we were able to identify the specific, small-to-moderate improvements relating specifically to the use of the accentuated eccentric loading used by AEL.

Accentuated eccentric loading was advantageous compared to traditional isoinertial loading for improving maximum isometric and eccentric force production. Greater increases in isometric force production were evidenced by the statistically greater improvements in isometric knee extension torque compared to CON and greater effect size (*g* = 0.95 vs. 0.49) compared to TRAD over 10 weeks (Figure 3), as well as the improvements in force production specifically during the last 5 weeks of training (AEL = ~7%, *p* = 0.03, *g* = 0.33; TRAD = ~−1%, *p* = 0.58, *g* = −0.06). In this regard, the first 5 weeks of training likely provided a more optimal training environment for both TRAD and AEL subjects to improve their performance level, whilst the additional benefit for AEL was visible in the second 5-week mesocycle. These data indicate that benefits of accentuated eccentric loading in already-trained individuals may take some time to manifest and, therefore, that short periods of training (e.g., several sessions) may be insufficient to elicit meaningful improvements. It would be of interest to examine adaptations to even longer training periods in future to determine whether continued improvements can be elicited by accentuated eccentric loading or whether the continuous high intensity may lead to over-reaching.

Importantly, a significantly greater improvement in maximum isokinetic concentric torque was observed in AEL than CON (Figure 3A), which is consistent with the findings of Hortobágyi and DeVita (2000) and Hortobágyi et al. (2001). While some studies have shown improvements in concentric performance following eccentric-only training (Paddon-Jones et al., 2001; Seger and Thorstensson, 2005; Franchi et al., 2015), some previous studies have shown little adaptation of concentric force production (e.g., Komi and Buskirk, 1972; Hortobágyi et al., 1996; Higbie et al., 1996), which may not be an ideal outcome for certain populations where (functionally important) concentric force production is imperative. Consequently, it may be considered that training with accentuated eccentric loads is efficacious for the improvement of all contraction modes in previously strength-trained men.

Also, it is important to note that accentuated eccentric load training elicited an improvement in the ability to perform consecutive muscular contractions against a load of 75% 1-RM (i.e., fatigue resistance). This was evidenced by the statistically significant improvement in the unilateral knee extension repetition-to-failure test (~28%, *p* = 0.022, *g* = 0.73), whereas the change was at the level of a trend in TRAD (~24%, *p* = 0.076, *g* = 0.71). These data suggest that accentuated eccentric load training led to more systematic improvements in repeated force production whereas there was likely greater variability in TRAD, which resulted in a lack of statistical significance. As the present study did not aim to match workload in the training sessions, in contrast to other studies where no difference in performance improvement was observed (e.g., Brandenburg and Docherty, 2002), this finding could reflect the greater overall work required when using accentuated eccentric loading protocols. Increased repetition-to-failure performance was also observed in a group performing greater workload due to the use of variable external resistance vs. constant external resistance in a recent study (Walker et al., 2013). An increase in the ability to complete a high volume of work prior to failure might be important in that a greater workload can be completed in each training session, which may provide a greater ongoing stimulus for increasing strength and muscle mass. It may also indicate that the training offers a method to improve fatigue resistance that may benefit performance in activities of daily living or sporting activities.

It should be noted that the final sample size in the present study (*n* = 10+10+8) was likely statistically underpowered to detect differences, particularly between the intervention groups. Results of our priori suggested that sample sizes of 11–12 per group would be sufficient for a power of 0.8. Consequently, due to difficulties in recruiting trained subjects to perform the study in addition to the number of drop-outs and magnitude of strength improvements during the intervention (+10–30% rather than our expected +5–15%) probably increased the likelihood of type II errors. Nevertheless, the observed differences between AEL and CON, as well as the greater effect sizes in AEL compared to ISO give confidence in our interpretation that training with accentuated eccentric loads induced greater strength gains in trained individuals despite the potential under powering of the present study. Furthermore, the small differences between training with traditional isoinertial and accentuated eccentric loads combined with limited sample sizes may have led to other studies being unable to determine differences between groups (Nichols et al., 1995; Norrbrand et al., 2008; Raj et al., 2012; Dias et al., 2015).

There are several potential mechanisms that may have contributed to the observed improvements in maximum force production in AEL in the present study. VL and VM CSA was significantly increased in both AEL and TRAD (~11–16%) over the 10-week training period, and these increases were statistically greater than CON (in which no change was observed). The increases observed in AEL and TRAD were within the typical range reported previously (Wernbom et al., 2007). These increases are notable given that the subjects were accustomed to resistance training and the exact values may have been influenced to some extent by variance inherent in the measurements (CV%; 4–10%). Nevertheless, at least in the short-term, accentuated eccentric load training does not provide a greater benefit than that provided by the imposition of a supervised, high intensity training program with post-exercise protein ingestion. Furthermore, the increases in lean leg mass (assessed by DXA) strengthen the interpretation that both training protocols were equally effective in eliciting muscle hypertrophy in the intervention groups. These findings are consistent with those of Friedmann-Bette et al. (2010) who observed similar increases in quadriceps CSA after 6 weeks of traditional isoinertial loading (~8%, according to their Figure 3) and accentuated eccentric loading (~6%) in power athletes under supervision. This is also consistent with the proposal that, as long as sets are performed to concentric failure, small differences in loading intensity of the two intervention groups in the present study may not impact protein synthesis (Burd et al., 2012). Previous data that did show a slight advantage from training with accentuated eccentric loads over traditional isoinertial training for muscle hypertrophy (Friedmann et al., 2004; Norrbrand et al., 2008) were conducted in previously untrained individuals. It may be that training status is the cause of this disparity between studies, rather than training intensity, as Friedmann et al. (2004) used low loads (~30% 1-RM) while Norrbrand et al. (2008) used 7-RM loads. Collectively, such results indicate that additional hypertrophy may not be elicited by accentuated eccentric loading in well-trained individuals over a 10-week training period (at least using training volumes of ≤ 60 repetitions per session per muscle group) and highlights the importance of studying the effect of resistance training protocols in well-trained subjects.

Another potential adaptation that may have underpinned the increase in strength elicited by training with accentuated eccentric loads is an improvement in muscle activation. Related to this, absolute surface EMG_RMS_ measured during maximum eccentric (isokinetic) and isometric knee extension increased significantly in the AEL group only. Interestingly, previous studies (Komi and Buskirk, 1972; Hortobágyi et al., 1996) have observed greater increases in EMG amplitude following eccentric-only vs. concentric-only resistance training. It has been suggested that, due to the muscle damaging nature of eccentric actions, motor unit recruitment and synchronization are altered during recovery from high intensity eccentric exercise (Dartnall et al., 2011) and this may affect EMG amplitude. We have examined this possibility *post-hoc* with quantification of mean and median spectral frequencies but observed no changes (e.g., AEL median power frequency = 85.3 ± 7.7 to 88.8 ± 9.4, *p* = 0.15), indicating that any residual effects of the final training bout had subsided prior to testing.

It is well accepted that there are several factors that affect surface EMG signals (i.e., affecting both maximum peak-to-peak M-wave and voluntary EMG amplitude) that are independent of motor unit activity (Farina et al., 2004). In the present study, significant increases were observed in both voluntary EMG and M-wave amplitudes in AEL (consequently no changes occurred in EMG:M-wave ratio). Therefore, one explanation for the increased EMG activity in AEL is that peripheral (i.e., muscular) factors had a greater effect on the EMG signal than neural (i.e., central) factors (Arabadziev et al., 2014), and that these affected both M-wave and voluntary EMG amplitudes. As there were no changes in spectral frequencies it would appear that there were no systematic changes in the EMG electrode placement relative to the innervation zone or tendon but, for example, lesser amplitude cancelation of the EMG signal and/or changes in the propagation of action potentials may have led to an increased EMG_RMS_ of similar magnitude to the increase in the maximum M-wave amplitude. There is some experimental evidence to suggest that this can occur during relatively slow, submaximal-load training (Maffiuletti and Martin, 2001). Another possibility is there were changes in alignment between muscle fascicles or subcutaneous fat and the EMG electrodes. We assessed these possibilities *post-hoc* through the acquired ultrasound images and observed some indication that changes in fascicle angle relative to the electrode could have partly influenced EMG signals in AEL (change in VL fascicle-to-electrode angle to M-wave; *r* = 0.33, *p* = 0.39, *n* = 9 and change in VL fascicle-to-electrode angle to VL EMG amplitude; *r* = 0.72, *p* = 0.043, *n* = 8). Nevertheless, a more detailed assessment of motor unit discharges and conduction velocities, as well as potential peripheral causes of altered EMG signals could be considered in future studies.

Despite the difficulty in interpreting the changes in surface EMG signals, a significant increase in quadriceps' voluntary activation level (VA%) was observed exclusively in AEL, as measured by the twitch interpolation technique during maximal isometric actions. This change was particularly evident in the second 5-week mesocycle and is considered to indicate an improved volitional drive to the muscle (Knight and Kamen, 2001), which may be due to increased firing frequency. This method may have allowed more specific assessment of muscle activation than that provided by surface EMG, and the findings are in-line with the greater improvements in force production during this period in AEL. While there were no between-group differences, the twitch interpolation technique has been reported to be insensitive to detect changes and a mean increase of 3.5% could be considered meaningful (Herbert and Gandevia, 1999). Unfortunately, measurements were not made during concentric and eccentric actions in the present study because of the likelihood of fatigue associated with numerous maximal contractions; it could, therefore, be considered in future studies. One hypothesis explaining the increase in neural drive after accentuated eccentric loading is that neural inhibition may have been reduced during maximum eccentric (Aagaard et al., 2000) and isometric actions. However, it cannot be fully discounted that peripheral factors (e.g., altered calcium concentrations), which have not currently been determined in the literature, may have also influenced the increase in VA% (Gandevia, 2009). Nevertheless, the increased EMG_RMS_ and VA% was uniform in those muscle actions where AEL demonstrated greater strength gains than TRAD (i.e., eccentric and isometric) and in which there were no changes in TRAD for EMG_RMS_ and VA%. Hence, it remains possible that the mechanism(s) responsible for these neurophysiological findings may also have contributed to the larger strength improvements in AEL.

In conclusion, accentuated eccentric loading seems to provide an additional training stimulus to increase maximum force production, as well as increasing work capacity/reducing fatigue during lifting in previously trained subjects. That the improvements in AEL were greater than TRAD in the second 5-week mesocycle indicates that these changes may take several weeks to manifest in subjects accustomed to resistance training when the addition of supervision/motivation, greater loading intensities, and assistance at concentric failure are made. Nevertheless, both the traditional isoinertial and accentuated eccentric loading training programs were equally effective in eliciting increases in muscle CSA in subjects accustomed to resistance training. Therefore, mechanisms other than muscular hypertrophy, including increases in muscle activation, appear to underpin the greater improvements following training with accentuated eccentric loads.

## Author contributions

Conceived and designed the experiments: SW, AB, GH, RN, KH. Performed experiments: SW, GH, JT. Analyzed data: SW, AB, GH. Interpreted results of research: SW, AB, GH, RN, KH. Drafted, edited, critically revised paper, and approved final version of manuscript: SW, AB, GH, JT, RN, KH.

## Funding

This work was joint-funded by the Department of Biology of Physical Activity, University of Jyväskylä, Finland and the Centre for Exercise and Sports Science Research (CESSR), Edith Cowan University, Australia.

### Conflict of interest statement

The authors declare that the research was conducted in the absence of any commercial or financial relationships that could be construed as a potential conflict of interest.
